# Understanding the influence of the COVID-19 pandemic on hospital-based mortality in Burundi: a cross-sectional study comparing two time periods

**DOI:** 10.1017/S0950268820002770

**Published:** 2020-11-13

**Authors:** D. Habonimana, L. Ouedraogo, E. Ndirahisha, N. Misago, R. Ciza, D. Niyomwungere, F. Niyongabo, J. B. Irakoze, J. D. Nkurunziza, S. Manirakiza

**Affiliations:** 1 Research and Innovation Unit, Department of Community Medicine, Faculty of Medicine, University of Burundi, Bujumbura, Burundi; 2 Regional Adviser for Sexual and Reproductive Health, World Health Organization Regional Office for Africa, Brazaville, Congo; 3 Department of Internal Medicine, Faculty of Medicine, University of Burundi, Bujumbura, Burundi; 4 Health Healing Network Burundi, Bujumbura, Burundi; 5 National Health Institute, Ministry of Public Health and AIDS Control, Bujumbura, Burundi; 6 Factulty of Medicine, University of Burundi, Bujumbura, Burundi; 7 Department of Statistics, Higher Institute of Education, Bujumbura, Burundi; 8 Department of Radiology and Imaging, Faculty of Medicine, University of Burundi, Bujumbura, Burundi

**Keywords:** Burundi, COVID-19, hospital-based deaths, tertiary hospital

## Abstract

This study used hospital records from two time periods to understand the implication of COVID-19 on hospital-based deaths in Burundi. The place of COVID-19 symptoms was sought among deaths that occurred from January to May 2020 (during the pandemic) *vs.* January to May 2019 (before the pandemic). First, death proportions were tested to seize differences between mortality rates for each month in 2020 *vs.* 2019. In the second time, we compared mean time-to-death between the two periods using the Kaplan–Meier survival curve. Finally, a logistic regression was fitted to assess the likelihood of dying from COVID-19 symptoms between the two periods. We found statistical evidence of a higher death rate in May 2020 as compared to May 2019. Moreover, death occurred faster in 2020 (mean = 6.7 days, s.d. = 8.9) than in 2019 (mean = 7.8 days, s.d. = 10.9). Unlike in 2019, being a male was significantly associated with a much lower likelihood of dying with one or more COVID-19 symptom(s) in 2020 (odds ratio 0.35, 95% confidence interval 0.14–0.87). This study yielded some evidence for a possible COVID-19-related hospital-based mortality trend for May 2020. However, considering the time-constraint of the study, further similar studies over a longer period of time need to be conducted to trace a clearer picture on COVID-19 implication on hospital-based deaths in Burundi.

## Introduction

Despite global efforts to contain the novel coronavirus disease 2019 (COVID-19) pandemic, most countries continue to register unprecedented death rates [1, 2]. As of 18 July 2020, the World Health Organization (WHO) reported 13.9 million confirmed cases globally and nearly 600 000 deaths due to the virus [3]. Although Asia, Europe and America remain the most affected continents, the disease has spread to 216 countries so far, including those in Africa [3].

In Africa, the first COVID-19 confirmed case was reported on 27 February 2020 [3]. Within 1 month, nearly 3000 cases and more than 400 deaths were registered [3]. As of mid-July 2020, the total number of cases had reached half a million with 20 000 deaths [3]. By then, among most affected African countries included South Africa (337 594 confirmed cases and 4804 deaths), Egypt (86 474 confirmed cases and 4188 deaths), Nigeria (35 454 confirmed cases and 772 deaths) and Ghana (26 572 confirmed cases and 144 deaths) [3-5]. In some African countries such as Burundi, however, the pandemic seemed to progress at a much slower pace as compared to the worldwide trends.

**Fig. 1.**
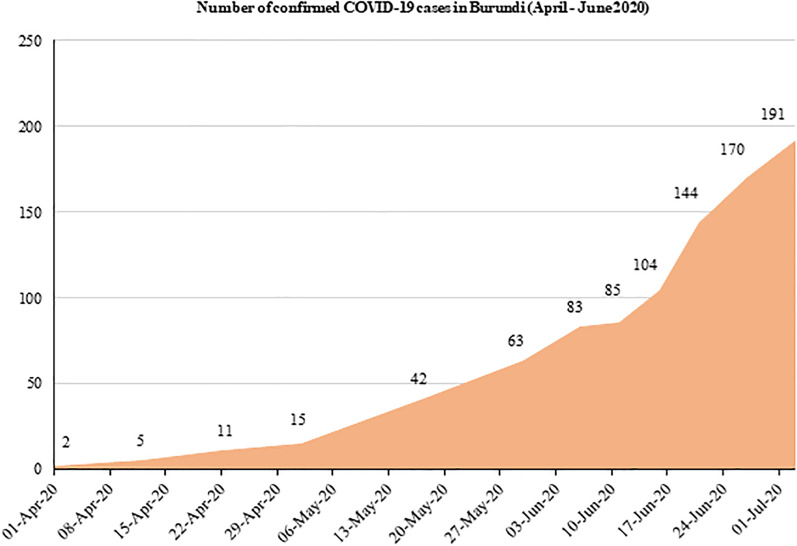
Trend in total number of confirmed COVID-19 cases in Burundi.

Burundi, a country located astride Eastern and Central Africa, announced the first two COVID-19 confirmed cases on 31 March 2020. Following the announcement, there has been a slow but continuous upward trend in the number of new infections reported in the country [6]. Figure 1 shows that new COVID-19 confirmed cases increased from 2 to 191 individuals within a period of 3 months. However, until late July 2020, only one COVID-19-related death had been officially reported earlier on 13 April 2020 [7, 8]. In this country, uncertainty about the real COVID-19 situation remains.

Our study sought to understand the place of COVID-19 on hospital-based deaths in Burundi as of May 2020. Specifically, the study aimed to detect the existence of a difference in crude hospital-based death rates by comparing two periods before and during the pandemic (January to May 2019 *vs.* January to May 2020). Furthermore, by comparing results between periods, the study sought to seize the likelihood of dying from COVID-19 symptoms or any of the underlying health conditions associated with COVID-19 mortality [9–11].

## Methods

### Study description

We employed a cross-sectional comparative study using two different time periods: January to May 2019 (period before the COVID-19 pandemic) and January to May 2020 (period during the COVID-19 pandemic). The study was conducted in Kamenge Medical Teaching Hospital which is a tertiary hospital of the University of Burundi. Based on the socio-demographic characteristics associated with COVID-19 mortality worldwide, the study used data from internal medicine and intense care unit where the vast majority of COVID-19 patients are admitted [12, 13]. In total, 351 deaths of which 181 occurred between January and May 2019 and 170 between the same period in 2020 were included in the analysis.

### Data collection

Data were collected using hospital records. We collected the crude numbers of hospital admissions and deaths monthly in each study period as summarised in Table 1. For each registered death, we collected socio-demographic information including age, sex, weight, height, pregnancy, place of residence, profession, date of admission and date of death as well as clinical information on COVID-19 symptoms including fever or chills, dry cough, tiredness, muscle or body aches and pains, sore throat, headache, new loss of taste or smell, congestion or runny nose, nausea or vomiting, difficulty in breathing or shortness of breath, chest pain or pressure and loss of speech or movement. Additionally, information on underlying health conditions including hypertension, asthma, diabetes, heart disease, kidney failure, chronic obstructive pulmonary disease and tuberculosis which are associated with the increased risk of COVID-19 morbidity and mortality was collected [9–11].

**Table 1.** Number of admissions and deaths per study period

	2019		2020	
	Admissions	Deaths: *n* (%)	Admissions	Deaths: *n* (%)
January	206	43 (20.9)	218	24 (11.0)
February	190	37 (19.5)	203	28 (13.8)
March	220	37 (16.8)	205	47 (22.9)
April	216	36 (16.7)	210	27 (12.9)
May	206	28 (13.6)	201	44 (21.9)
Total	1038	181 (17.4)	1037	170 (16.4)

### Statistical analysis

Data analysis was done in three steps. In the first step, monthly death rates were plotted on a line graph to observe mortality trends between the study periods. A test of proportion was then used to test statistical differences between mortality rates for each month in 2019 *vs.* 2020. A 95% *P*-value was used to ascertain significance of the differences. In the second step, a Kaplan–Meier survival curve was fitted to observe the graphical trend and determine the difference in mean time from the date of hospital admission to the date of death between the study periods. The final step involved fitting two separate logistic regression models for the two time periods. For each time period, we created a binary outcome ‘COVID-19 symptom’ taking value 0 if patient *i* (who died) presented one or more symptom(s) of COVID-19 and value 1, otherwise. A logistic model was then constructed as below:
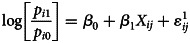


The outcome variable is the log odds that patient *i* died from alterative *j* relative to alternative 0, where 0 means presenting one or more COVID-19 symptom(s); and 1 implies dying without a COVID-19 symptom. Predictors are represented by a standard vector of covariates ***X***. They include the patient's age, sex, body mass index (BMI), residence, profession and underlying health conditions associated with COVID-19 mortality. The model includes *β*_0_ which captures fixed effects and *β*_1_ which detects random effects on the probabilities of dying from one or more COVID-19 symptom(s). A 95% confidence interval (CI) was used to ascertain significance of predictors; which the literature claims to be more reliable for discrete models [14, 15].

## Results

### Demographic and clinical characteristics of deaths

Overall, majority of deaths occurred among older people, those with higher body mass indices and among individuals without a profession. As shown in Table 2, there was no evidence of difference in mortality rates between 2019 and 2020 by age, BMI, residence, COVID-19-related symptoms and underlying health conditions [9–11].

**Table 2.** Demographic and clinical characteristics

Variable	Overall *n* (%)	Year 2019 *n* (%)	Year 2020 *n* (%)	*Z* value	95% *P*-value
Age					
Up to 15 years old	25 (9.4)	12 (8.5)	13 (10.4)	−0.5291	0.5967
16 to 50 years old	116 (43.7)	62 (44.0)	54 (43.2)	0.1310	0.8958
51 to 70 years old	74 (27.9)	38 (26.9)	37 (29.6)	−0.4876	0.6258
Older than 70 years	50 (18.8)	29 (20.6)	21 (16.8)	0.7896	0.4297
Sex					
Female	137 (48.4)	68 (48.2)	66 (53.2)		
Male	146 (51.6)	73 (51.8)	58 (46.8)	0.4175	0.6763
BMI					
Up to 18	37 (21.4)	16 (21.6)	21 (21.2)	0.0635	0.9494
19 to 25	101 (58.4)	48 (64.9)	53 (53.5)	1.5050	0.1323
Above 25	35 (20.2)	10 (13.5)	25 (25.3)	−1.9107	0.0560
Chronic condition^a^					
No	137 (48.4)	79 (49.7)	59 (47.2)	0.4175	0.6763
Yes	146 (51.6)	80 (50.3)	66 (52.8)		
Residence					
Bujumbura (capital city)	129 (48.9)	74 (52.9)	55 (44.0)	1.4439	0.1488
Other	135 (51.1)	66 (47.1)	70 (56.0)		
Profession^b^					
No	159 (62.6)	92 (69.2)	68 (55.7)	2.2229	0.0262
Yes	95 (37.4)	41 (30.8)	54 (44.3)		
COVID-19 symptoms^c^					
Yes	113 (39.9)	59 (37.1)	54 (43.6)		
No	170 (60.1)	100 (62.9)	70 (56.4)	1.1077	0.2680

a Living with either one or more of the following health conditions: hypertension, asthma, diabetes, heart disease, kidney failure, chronic obstructive pulmonary disease and tuberculosis which have been found to predict high likelihood of dying from COVID-19.

b Household wives and farmers were classified as ‘having no profession’.

c Symptoms suggestive for COVID-19 included fever or chills, dry cough, tiredness, muscle or body aches and pains, sore throat, headache, new loss of taste or smell, congestion or runny nose, nausea or vomiting, difficulty breathing or shortness of breath, chest pain or pressure and loss of speech or movement.

### Hospital-based mortality rates

**Fig. 2.**
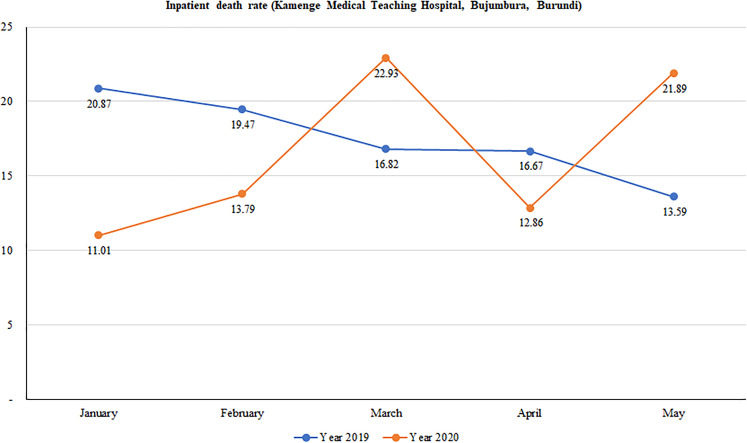
Trend in hospital-based mortality rates at Kamenge Medical Teaching Hospital, Bujumbura, Burundi.

We found a wide variation in mortality rates between January and May in 2019 and in 2020. Overall, although death rates continuously decreased over time in 2019, there was a considerable variation in death rates in 2020 with peaks observed in March and again in May. Figure 2 shows that mortality rates dropped from 20.9% in January to 16.8% in March and further declined to 13.6 in May 2019. Conversely, mortality rates sharply increased from 11.0% to 22.9% from January to March of 2020; halved in April (12.9%) before doubling in May (21.9%).

Statistical differences in mortality rates between 2019 and 2020 are summarised in Table 3. We found significant evidence of a higher death rate registered in January of 2019 than January of 2020 (20.9% *vs.* 11.0%; *P*-value = 0.005). Contrary to this, a higher death rate was observed in May of 2020 than in May of the previous year (21.9% *vs.* 13.6%; *P*-value = 0.028). There was no significant difference between death rates in 2019 and 2020 for the months of February, March and April.

**Table 3.** Results from the tests of proportions

	2019		2020		*Z* value	95% *P*-value
	Admissions	Deaths: *n* (%)	Admissions	Deaths: *n* (%)		
January	206	43 (20.9)	218	24 (11.0)	2.7821	0.0054
February	190	37 (19.5)	203	28 (13.8)	1.5146	0.1299
March	220	37 (16.8)	205	47(22.9)	−1.5805	0.1140
April	216	36 (16.7)	210	27 (12.9)	1.0982	0.2721
May	206	28 (13.6)	201	44 (21.9)	−2.1940	0.0282
Total	1038	181 (17.4)	1037	170 (16.4)	0.6379	0.5235

**Fig. 3.**
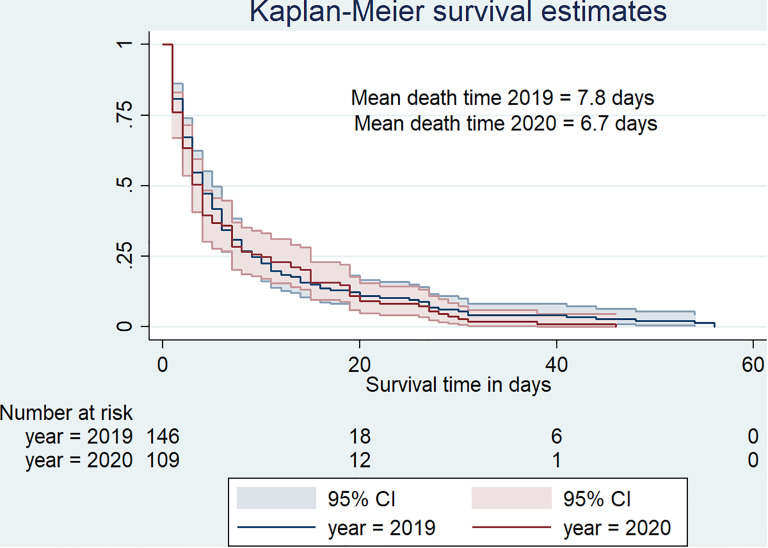
Survival curve for hospital-based death duration from January to May 2019 versus January to May 2020.

Kaplan–Meier curves indicate that death was quicker in 2020 than in 2019 (Fig. 3). In fact, deaths occurred in 6.7 days (s.d. = 8.9, median = 3) in 2020 against 7.8 days (s.d. = 10.9, median = 4) in 2019.

### Determinants of hospital-based mortality with COVID-19 symptoms

Results from the logistic model are summarised in Table 4. In 2019, we did not detect significant predictors of death with one or more COVID-19 symptom(s). Contrarily to 2019, being a male was significantly associated with a much lower likelihood of dying with one or more COVID-19 symptom(s) in 2020 (odds ratio (OR) 0.35, 95% CI 0.14–0.87). Equally, deaths with one or more COVID-19 symptoms in 2020 were about 66% less likely among those with employment compared to those without employment. Similar to 2019, age, BMI, underlying health conditions and residence were not significant predictors of death with COVID-19 symptoms in 2020.

**Table 4.** Determinants of hospital-based deaths in 2019 *vs.* 2020

Outcome = deaths with or without COVID-19 symptoms Base = deaths without COVID-19 symptoms				
	2019		2020	
	OR	95% CI	OR	95% CI
Age *(reference* *=* *up to 15 years old)*				
16 to 50 years old	3.59	0.27–47.35	0.92	0.15–5.75
51 to 70 years old	1.39	0.11–17.82	0.67	0.08–5.27
Older than 70 years	2.13	0.11–40.34	1.07	0.15–7.74
Sex *(reference* *=* *female)*				
Male	3.78	0.90–15.89	0.35	0.14–0.87
BMI *(reference* *=* *up to 18)*				
19 to 25	3.30	0.72–15.04	0.82	0.22–3.06
Above 25	1.70	0.24–12.09	0.59	0.13–2.74
Chronic condition *(reference* *=* *no)*				
Yes	1.10	0.32–3.76	1.54	0.59–3.97
Residence *(reference* *=* *Bujumbura)*				
Other	0.77	0.23–2.55	0.68	0.26–1.79
Profession *(reference* *=* *no)*				
Yes	1.51	0.36–6.32	0.34	0.12–0.94

## Discussion

The aim of this study was to understand the implications of COVID-19 on deaths occurring in a tertiary hospital located in Bujumbura, Burundi. The study used aggregated hospital data to determine whether COVID-19 was associated with an increase in hospital-based mortality rates. Furthermore, the study used individual clinical data to investigate the place of COVID-19 symptoms and underlying health conditions in hospital-based deaths occurring during the pandemic.

With respect to hospital-based mortality rates, we did not find evidence that the disease caused mortality rates to increase compared to the previous year. There was a wide variation in death rates, very difficult to attribute to the pandemic. Unlike in most countries around the world, hospital-based mortality rates under COVID-19 circumstances did not increase rapidly in Burundi. However, from the time Burundi confirmed new COVID-19 cases (31 March 2020), there seemed to be an interesting trend. With a hospital death rate of nearly 13% in April, the figure sharply rose to about 22% in the following month. Most importantly, our findings suggested that mortality rate became significantly higher in May 2020 which could be considered as an onset of the pandemic in Burundi. Additionally, the study found evidence that time-to-death was much shorter in 2020 with patients dying in 6.7 days on average. Therefore, a combination of the above evidence i.e. date of appearance of COVID-19 new cases, sharp increase in mortality rate in the following month (May 2020), and the shorter time-to-death observed in the same period could serve as an initial warning of COVID-19 impact in the country. Similar warning signs have been reported in many COVID-19-affected countries around the world [16–18].

Results from the logistic regression showed a much lower likelihood of dying with one or more COVID-19 symptom(s) among males and patients with a profession. Despite convincing results from a recent study on 26 countries by the World Bank in which the author found that a greater share of reported COVID-19 deaths occurred at younger ages in low- and middle-income countries [19], our study showed neutral stand with that regard. However, it is important to note that the above evidence seems to contradict a wealth of literature on the vulnerability of older people to COVID-19. For instance, in a recent study published in *The Lancet Infectious Diseases*, Verity *et al*. found the death rate from COVID-19 to be 7.8 among people aged over 80 years and declined with age to 0.0016% in children aged 9 years and below [20]. Also, it has been found that the incidence of severe COVID-19 cases requiring hospital care increases with age [17]. In our study, we also found significant evidence that the likelihood of dying from one or more COVID-19 symptoms decreased with male sex and profession. This finding contradicts that of the World Bank in their study conducted on data from 26 countries which found a male vulnerability [19]. Male vulnerability was also found in China [21]. Similarly, in Italy and in the USA, the COVID-19 fatality rate was equally higher in men. In the April 2020 report from the Italian National Institute of Health, of 23 188 deaths from COVID-19 infection, approximately 70% were males [22]. In the USA, death counts for COVID-19 between February and April 2020 indicated a similar male vulnerability (59% male deaths of 37 308 deaths) [22–24].

Altogether, our study yielded some evidence for a possible COVID-19-related hospital-based mortality trend for May 2020. However, this evidence needs to be treated with caution as the study has some limitations. On the one hand, our study was conducted over a period of 5 months of which only 2 months were after the confirmation of new COVID-19 cases in Burundi. Therefore, we would acknowledge the time-constraint and recommend that similar studies be conducted using data collected on a longer period of time to detect a much clearer picture of COVID-19 trend. Equally important to mention is the use of a secondary data source which inevitably contains little information compared to needed data. On the other hand, our study controlled for time by comparing two time periods with and without COVID-19 contexts, which is an asset to highlight. Most importantly, by comparing exactly similar months of 2020 and 2019, the study aimed to control for potential confounders (i.e. seasonal causes of mortality, health policy, change in practice, innovative interventions, etc.) which could have a causal relationship with hospital-based deaths (other than the COVID-19 crisis). To further strengthen the evidence, the study modelled deaths to COVID-19 suggestive symptoms alongside known underlying health conditions that increase the risk of mortality from the disease.

## Conclusions

Although the country has reported one COVID-19-related death as of May 2020, there seemed to be evidence that COVID-19 made a potential contribution to deaths occurring in May 2020 in Kamenge Medical Teaching Hospital of Bujumbura, Burundi. Despite the evidence being partial, the study generated some evidence of the potential implication on hospital-based deaths. Owing to the study limitations such as time constraints which did not allow collection of longitudinal data, authors of this study recommend that similar studies be conducted for a longer period of time. As such, a clearer picture of hospital-based mortality as well as predictors associated with deaths would help to conclude on the subject matter.

## Data Availability

The data and dofiles that support the findings of this study are available from the corresponding author and can be obtained by request. E-mail: desire.habonimana@ub.edu.bi.
